# Psychological differences and similarities between vegans, prospective vegans, and vegetarians. Motivation, knowledge, vegan literacy – and cheese

**DOI:** 10.3389/fpsyg.2024.1163869

**Published:** 2024-04-10

**Authors:** Roland Mayrhofer, Lara M. Roberts, Julia M. Hackl, Katja Frischholz

**Affiliations:** Department of Psychology, University of Regensburg, Regensburg, Germany

**Keywords:** vegan, vegetarian, sustainable diet, ecological motivation, animal motivation, food literacy, food knowledge

## Abstract

**Introduction:**

Although vegan and vegetarian diets and lifestyles differ significantly from each other, among other things, notably in their respective consequences regarding animal welfare and their ecological impact, vegans and vegetarians are often grouped together and usually compared to omnivores in psychological research. Considering that vegans and vegetarians often share similar motives for their lifestyle choices, namely animal and environmental issues, the question arises why similar motives lead to different conclusions and correspondingly different behaviors, most notably, of course, that vegetarians consume animal-derived foods such as cheese or milk while vegans do not consume animal-derived products (e.g., food, cosmetic products). This is why this study explored the psychological differences between vegans, vegetarians, and prospective vegans – the latter group being located in an intermediate, transitionary position. Focusing on the motivational, affective and cognitive components of dietary transition and participants’ adherence to eating patterns, reasons for said patterns, possible hinderances to becoming vegan, the role of participants’ social environments, and the impact of various misconceptions regarding the feasibility of a vegan diet in everyday life were all explored.

**Methods:**

An observational study was conducted via online questionnaire (1420 participants).

**Results:**

Significant differences were found between vegans, prospective vegans, and vegetarians, especially concerning their knowledge of issues pertaining to their respective lifestyles.

**Discussion:**

The critical role of knowledge is invoked as an explanation as to why vegans and vegetarians display different behaviors although they share a similar motivation. Thus, in this study the concept of vegan literacy is introduced. Additionally, the distinctive role of cheese is explored, discussing possible indications of its potentially addictive nature and, consequently, the importance of cheese as a hindering factor for pursuing a vegan diet.

## 1 Introduction

Besides many animal rights and animal welfare issues, diet has a major impact on both the individual’s ecologic footprint and health. There is substantial evidence supporting the fact that animal products and animal-based foods have a negative impact on the environment, and this evidence includes, for example, the related increased emission of greenhouse gasses (e.g., Notarnicola et al., 2017; Chai et al., 2019; Rabès et al., 2020; Allenden et al., 2022; Filippin et al., 2023; McDougall, 2023; Scarborough et al., 2023; see, however, also Rosi et al., 2017; Vettori et al., 2021; Dorgbetor et al., 2022). Similarly, vegan diets are associated with potential health benefits (e.g., Wirnitzer, 2020; Selinger et al., 2022; McDougall, 2023). Seeing as diets based on less or no animal-derived products do not impact the environment, health, or animals in the same way as an omnivorous diet does, vegetarian and vegan diets are of interest to current research. For example, even small amounts of animal-derived food appear to increase the environmental impact of diets (Filippin et al., 2023), or, similarly, a vegan diet positively affects athletes and physically active people (Wirnitzer, 2020). Seeing as there are substantial differences between vegan and vegetarian diets, it may be fruitful to investigate said difference also for psychological issues. Although there is extensive research on various issues pertaining to veganism and vegetarianism, in psychological research, vegetarians and vegans are often grouped together (Rosenfeld, 2019). This mirrors how the omnivorous outlook is still considered the norm and also how deviations from a diet including meat are grouped together regardless of their differences. However, this does not reflect that there are fundamental differences between vegans and vegetarians regarding not only—on a more evident level—their eating patterns (Cherry, 2015) but also their beliefs about animal rights, animal welfare, health (Ruby, 2012), and their overall ideals (Rothgerber, 2015a; Weber and Kollmayer, 2022). Psychological, motivational, and behavioral differences and similarities between vegans and vegetarians are less explored so far (e.g., Kessler et al., 2016; Lund et al., 2016; Rosenfeld, 2019; Kirsten et al., 2020; Zaal et al., 2023). There is a lack of research on the psychological differences and similarities between vegans and vegetarians regarding motivation, knowledge regarding animal-related topics and/or diet, dietarian identity, and affectivity. Therefore, the aim of the present study was to investigate said issues, with a focus on the question which factors might contribute to the fact that there is a crucial difference between vegans and vegetarians, i.e., vegetarians consume animal products whereas vegans do not, although both groups share a similar motivation.

Usually, research in this area addresses either vegans or vegetarians. However, this study also includes prospective vegans—current vegetarians who are planning to become vegan—as they are located in an intermediate, transitionary position between a vegan and a vegetarian lifestyle, thereby illustrating the transition from vegetarian to vegan. To our knowledge, this is the first psychological study that aims to examine the abovementioned psychological aspects explicitly for prospective vegans, thus adding further dimensions to existing sociological findings (Waters, 2022). Taking prospective vegans into account is important for two reasons: First, from a research perspective, prospective vegans adopt an intermediate position between vegans and vegetarians, so psychological characteristics shown by both groups are most likely found in the group of prospective vegans. Therefore, a more differentiated analysis of said characteristics will be possible due to the data from this study. Second, given that a substantial number of vegetarians may potentially transition to a vegan diet—as shown by the results of this study—understanding prospective vegans will facilitate a better understanding of the psychological factors involved in the process of transitioning from a vegetarian diet to a vegan diet.

From a psychological perspective, it is important to address not only behavioral but also motivational and cognitive variables of the matter in question. Generally, eating behavior is a central part of peoples’ identity (Rosenfeld and Tomiyama, 2019; Markowski, 2023) and is subject to many motivational, cognitive, and affective processes. Regarding motivational processes, there are many similarities between vegans and vegetarians, and common motivations including health, the environment, and animals (Baroni et al., 2007; Janssen et al., 2016; North et al., 2021; Weber and Kollmayer, 2022). Additionally, a recent study adds another factor that may impact the choice of diet and lifestyle: personal accountability. Personal accountability is defined as a sense of consciousness for and an awareness of the environmental impact of one’s own pattern of consumption, and it may be a crucial motive in becoming vegan (Ghaffari et al., 2022). For example, personal accountability may be a factor when confronted with information about deforestation, loss of biodiversity (Poore and Nemecek, 2018), or anthropogenic greenhouse gas emissions caused by livestock farming (Tubiello et al., 2021).

Regarding cognition, the aspects dietarian identity and knowledge are relevant to the present study. Referring to dietarian identity, it seems that vegans perceive their diet as more closely linked to their identity than vegetarians (e.g., Rosenfeld, 2019). Concerning knowledge, there is extensive research on the relevance of knowledge in the process of *becoming* vegetarian and vegan (e.g., MacNair, 2001). However, there is a lack of data on the possible differences between vegans and vegetarians regarding knowledge.

Even though vegans and vegetarians are similar in many ways, it is important to note that there are also fundamental differences between these two groups. While vegetarians rely on many animal products as part of their lifestyle, veganism means “living without reliance on animal products” (Cherry, 2015, p. 56). Furthermore, while vegetarians often follow their diet for ethical reasons, veganism “represents an opposition to violent and exploitative human-nonhuman animal relations” (Cole and Morgan, 2011, p. 135), thus particularly highlighting ethical aspects. The differences between vegans and vegetarians also extend to ecological issues, seeing as environmental veganism includes “awareness about the negative ecological impact of the livestock industry, including the production of greenhouse gasses and deforestation” (Christopher et al., 2018, p. 55). Overall, it becomes increasingly evident how it does not adequately reflect the differences between vegetarians and vegans to group them together in (psychological) research. As Rothgerber (2015a, p. 255) states: “these two groups differed from each other more often than not.” However, many psychological mechanisms which differentiate vegans from vegetarians have not yet been researched in depth.

Emphasizing not only behavior but also mental processes and the prevailing mindset, research suggests that vegans are more consistent in following their ideals—fittingly, vegans hold stronger beliefs than vegetarians concerning the environment (Ruby, 2012; Rothgerber, 2015b) and animals, as suggested by the distinction between animal welfare and animal rights (e.g., Jena, 2017), and higher scores in idealism and empathy toward animals (Rothgerber, 2015a). However, vegetarians often state the same reasons as vegans as primary reasons for their lifestyle choices (e.g., Ruby, 2012) but at the same time appear to disregard the fact that a vegetarian lifestyle has a more negative impact on the environment and animals compared to a vegan lifestyle. Similarly, there are other ambiguities between vegans and vegetarians, e.g., even though many sorts of cheese are not even vegetarian (as cheese often contains rennet, which is derived from the stomach of calves; García-Gómez et al., 2021), vegetarians nevertheless often seem to assume that cheese only contains milk and additionally often falsely believe that no animals die as a consequence of their dietary choices. These examples suggest that specific knowledge is a crucial difference between vegans and vegetarians. This may also explain why their behavior differs, although both groups are motivated by similar reasons.

Beyond these cognitive variables, from a psychological viewpoint, it is necessary to also address motivation, knowledge, cultural differences, and potential challenges involved in various dietary patterns in the current debates on global warming or physical health. For this purpose, various frameworks with regard to veganism and vegetarianism have been developed in psychology. This further highlights the necessity to research in depth the mechanisms and processes within the human psyche, meaning in mind, motivation, and emotion which are relevant with regard to diet under the aforementioned vantage points.

Furthermore, social support and networks are crucial for sustaining a vegan or vegetarian diet (Haverstock and Forgays, 2012; Markowski and Roxburgh, 2019; Godin, 2023; Sirieix et al., 2023; Williams et al., 2023). Stigmatization and discrimination are hindering factors in both sustaining (Salehi et al., 2020) and transitioning to a plant-based diet (Markowski and Roxburgh, 2019). This stigmatization can occur on the part of the media, where veganism is frequently portrayed in a negative way and described as not being feasible in everyday life (Cole and Morgan, 2011; Brookes and Chałupnik, 2023) or in the form of stigmatization and prejudice on the part of close friends and family (Haverstock and Forgays, 2012; MacInnis and Hodson, 2017; Bolderdijk and Cornelissen, 2022). In addition, it is noteworthy that vegans report more criticism arising from their social environment due to their diet than vegetarians (Fiestas-Flores and Pyhälä, 2018). Moreover, high dietary adherence is associated with a strong perception that the vegetarian eating pattern is a central part of one’s identity (Rosenfeld and Tomiyama, 2019).

Overall, by investigating all of the abovementioned aspects—namely, emotions, motivation, cognition, knowledge, and social variables—the aim of the present study was to find out whether there are psychological differences between vegans, prospective vegans, and vegetarians and how said differences and similarities may be reflected in behavior, attitude, and knowledge.

## 2 Materials and methods

In this descriptive, cross-sectional study, a total of 1,776 participants completed an online survey in a 2-month period between October and December 2021. The participants took part voluntarily and on their own accord, responding to a request looking for vegans and vegetarians posted on social media in German-speaking groups on veganism and vegetarianism. There were no restrictions for participation. However, the following participants were excluded from the data analysis: all non-vegans and non-vegetarians,^1^ participants with missing or unclear data, e.g., leaving out questions (other than social demographic data), participants giving contradictory answers regarding their dietary pattern, participants who indicated that they did not process the survey meaningfully, or participants who were not located within the relative speed index (time-RSI) > 2 (see Leiner, 2019), which indicates that the survey was merely skimmed. Thus, after the exclusion of 356 cases, 1,420 participants were included in the final analysis.

This study was conducted online with no expected potentially negative effects on participants. Hence, no ethics approval was required according to German law. Participants were guaranteed anonymization of their data, according to the General Data Protection Regulation (GDPR) of the European Union, and all participants gave informed consent before participation.

First, data were collected on previous, current, and future dietary patterns, such as the duration of the current dietary pattern and reasons for current and future dietary patterns. Concerning the role of specific food types, participants were asked to indicate on a 5-point Likert scale (1 indicating “not difficult at all,” 5 “very difficult”) how difficult it actually was or would be to give up certain animal-derived food types.

Next, participants’ self-assessment regarding knowledge of diets, knowledge of the animal industry, and general assessment of the animal industry were measured on a scale from 0 (lowest) to 100 (highest). Subsequently, knowledge was assessed on the basis of McDonald’s (2000) concept of communicative knowledge, i.e., general knowledge about diets and the animal industry. Knowledge was measured with 19 items covering several key issues with regard to veganism, such as practices in the animal industry, knowledge about environmental aspects, and knowledge about diets. Participants were asked to indicate whether statements were correct or not, e.g., “Large areas of the tropical rain forests are cleared to grow soy, which is fed to livestock, especially cattle,” “In order to obtain the hormone PMSG used in pig breeding, several liters of blood are taken from pregnant mares every week,” or “A vegan diet inevitably contains too little protein sources and is therefore unhealthy in the long term” (reversed). There was also the option “I do not know” to prevent guessing, and some items were reversed to prevent acquiescence bias. To determine knowledge, the percentage of correct answers was calculated.

Participants also indicated which sources of information on vegan and vegetarian issues they use, e.g., books, scientific studies, or social media (see below) and how much time they spent on acquiring said information (hours per month: <1, 1–2, 3–4, 5–10, >10).

Finally, concerning attitude, instrumental knowledge, dietarian identity, and affectivity, the following measurements were taken: Participants’ attitude toward a vegan lifestyle was measured with 15 items which covered a variety of questions that are debated in the vegan community, e.g., “Leather is a by-product of meat production and therefore it is morally justifiable to buy products made from leather” (reversed), “Animals should also have fundamental rights,” and “Humanity is at the top of the hierarchy of living beings, so it is natural to use animals” (reversed). Agreement was measured with a 5-point Likert scale (1 indicating “do not agree at all,” 5 “fully agree”), and some items were reversed to prevent acquiescence bias. Instrumental knowledge, a concept also adopted from McDonald (2000), was measured with 14 items that covered a variety of issues faced by vegans in their daily life, e.g., “Buying vegan products would not be a problem for me,” “A vegan diet would be an extra time-consuming task for me” (reversed), or “When I read ingredient lists, I know which ingredients are not vegan.” Instrumental knowledge was measured with a 5-point Likert scale (1 indicating “do not agree at all” and 5 “fully agree”), and some items were reversed to prevent acquiescence bias. Dietarian identity was measured with the *Dietarian Identity Questionnaire* (Rosenfeld and Burrow, 2018), while the *Connectedness to Nature Scale* (Perrin and Benassi, 2009) provided items for affectivity with regard to animals and assessing to what extent participants were empathetic and connected to animals, covering both positive and negative emotions. Both dietarian identity and affectivity were measured with a 5-point Likert scale (1 indicating “do not agree at all” and 5 “fully agree”), and some items were reversed to prevent acquiescence bias. For attitude, instrumental knowledge, dietarian identity, and affectivity, the respective means were calculated from all items of the respective scales.

As the main goal of the present study was to investigate whether there are psychological differences between vegans, prospective vegans, and vegetarians concerning motivation, cognition, knowledge, emotion, and social variables, the mean values of the three beforementioned groups were compared using Analyses of Variance, the prerequisites of which were satisfactorily fulfilled. Significant results were further analyzed using Tukey-HSD *post-hoc* tests. Additionally, a linear regression was calculated to explore whether dietarian identity, attitude, affectivity, communicative knowledge, and instrumental knowledge can predict how long vegans have been following their dietary pattern.

## 3 Results

The sociodemographic data of the sample of the present study are given in Table 1.

**Table 1:** Sociodemographic data of the participants.

Variable	Vegan	Prospective vegan	Vegetarian
*N*	1,046	197	177
Mean age (SD)	37.73 (12.31)	32.03 (12.48)	30.12 (11.37)
Mean age at start of current dietary pattern (SD)	31.41 (11.85)	21.57 (11.09)	20.58 (10.29)
Mean duration (in years) of current dietary pattern (SD)	6.14 (5.91)	9.64 (9.47)	8.82 (8.21)
**Gender identity (%)**			
Female	80.8	87.3	82.5
Male	17.4	11.2	16.4
Other and not answered	1.8	1.5	1.1
**Residency (%)**			
Germany	87.2	86.3	92.1
Austria	9.2	9.6	6.2
Switzerland	2.1	1.0	0.6
Other and not answered	1.5	3.0	1.1
**Marital status (%)**			
Single	29.4	42.1	46.3
Relationship	34.3	34.5	30.5
Married	28.4	15.2	20.3
Widowed	0.9	0.5	0.6
Divorced	4.7	3.6	1.1
Other and not answered	2.3	4.1	1.1
**Religion (%)**			
Buddhism	1.5	1.5	0.6
Christianity	16.3	22.3	30.5
Hinduism	0.3	0	0
Islam	0.1	0.5	0.6
Judaism	0.2	0	0.6
Religious, unspecified	17.1	22.8	17.5
Not religious	60.1	48.2	49.2
Other and not answered	4.3	4.6	1.1
**Occupation (%)**			
Student	0.5	0.5	0.6
University student	16.5	41.6	48.0
Apprentice	1.8	3.6	1.7
Employed	68.5	41.1	45.2
Unemployed	2.0	2.5	0
Other and not answered	10.6	10.7	4.5
**Pets (%)**			
Yes	44.1	49.7	53.7
No	55.8	50.3	46.3
Not Answered	0.1	0	0
**Hours per month spent on information (%)**			
<1	6.8	9.1	19.2
1–2	18.1	33.5	42.9
3–4	28.9	29.4	24.9
5–10	21.6	14.2	9.0
>10	24.7	13.7	4.0

### 3.1 Motivation

**Figure 1:**
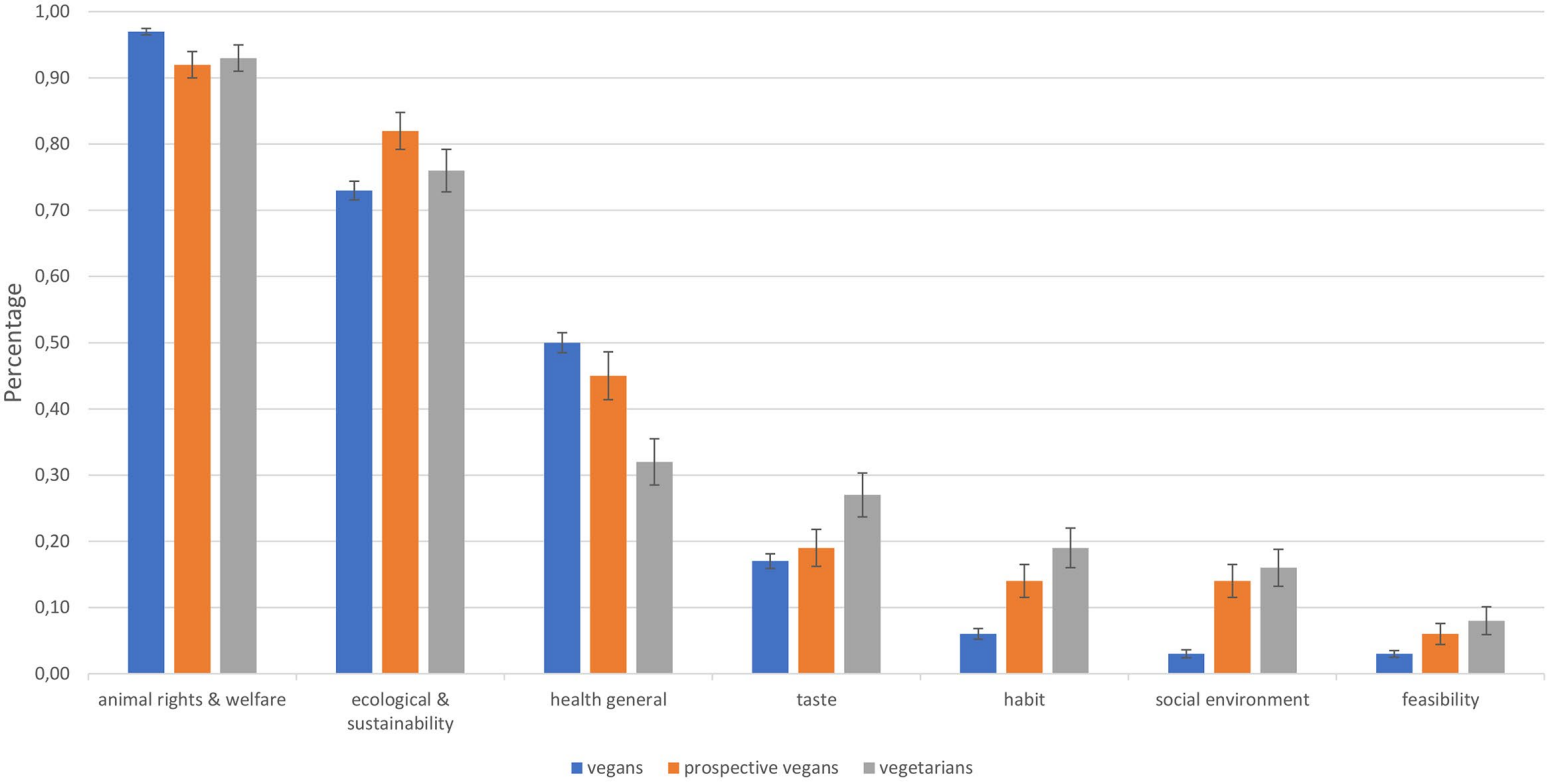
Reasons for current lifestyle: percentage of participants who stated the appropriate reason as motivation for their current lifestyle. Error bars represent the standard error of the mean.

The reasons for the current lifestyle are presented in Figure 1, represented as the percentage (dummy-coded) of how many participants stated the appropriate reason as motivation for their current lifestyle. Religious and spiritual reasons, specific medical needs, feasibility in daily life, losing or gaining weight, financial reasons, culture and tradition, and other reasons are not depicted in Figure 1 since there were no differences between groups regarding these reasons (see below).

While animal rights and welfare was the reason stated most often over all groups, followed by ecological and sustainability reasons and health reasons, a closer analysis of the respective percentages (dummy-coded) as provided by each group using a MANOVA revealed that there were significant differences in participants’ motivation regarding their current lifestyle between vegans, prospective vegans, and vegetarians for all reasons with the exception of specific medical needs (stated in total by 4.9%), religious and spiritual reasons (5.8%), gaining or losing weight (3.0%), and financial reasons (1.3%, all *p*s > 0.093). There were significant differences between the groups for the following reasons: animal rights and welfare: *F*(2, 1417) = 7.51, *p* = 0.001, η*_p_*^2^ = 0.010, ecological and sustainability: *F*(2, 1417) = 3.52, *p* = 0.030, η*_p_*^2^ = 0.005, health general: *F*(2, 1417) = 9.76, *p* < 0.001, η*_p_*^2^ = 0.014, taste: *F*(2, 1417) = 5.18, *p* = 0.006, η*_p_*^2^ = 0.007, habit: *F*(2, 1417) = 19.61, *p* < 0.001, η*_p_*^2^ = 0.027, social environment: *F*(2, 1417) = 30.70, *p* < 0.001, η*_p_*^2^ = 0.042, feasibility: *F*(2, 1417) = 8.49, *p* < 0.001, η*_p_*^2^ = 0.012.

Concerning prospective vegans’ reasons regarding their intention to become vegan, the three most prominent reasons were animal welfare and rights (stated by 80.7%), ecological reasons and sustainability (70.6%), and general health (26.9%). Other relevant reasons were taste (5.1%), social environment (5.1%), religious and spiritual reasons (3.0%), specific medical needs (3.0%), habit (1.5%), and feasibility in daily life (0.5%). Financial reasons as well as culture and tradition were not stated by any of the participants.

**Figure 2:**
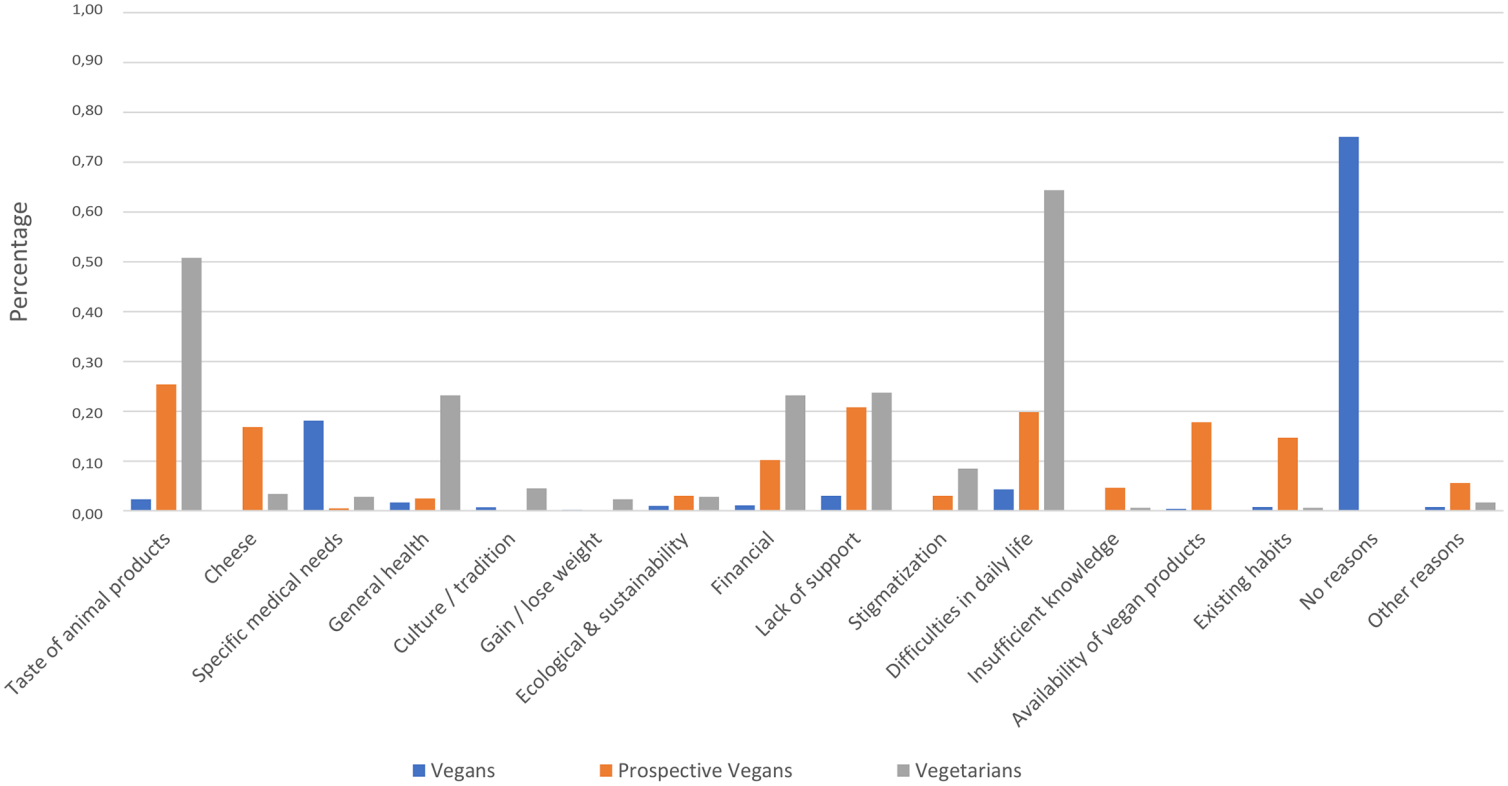
(Potential) Reasons hindering a vegan lifestyle: percentage of participants who stated the appropriate reason. Not all options were available for all groups.

Similarly, Figure 2 provides the reasons (potentially) hindering a vegan lifestyle, as given by vegetarians, prospective vegans, and vegans.

### 3.2 The role of specific food types

**Figure 3:**
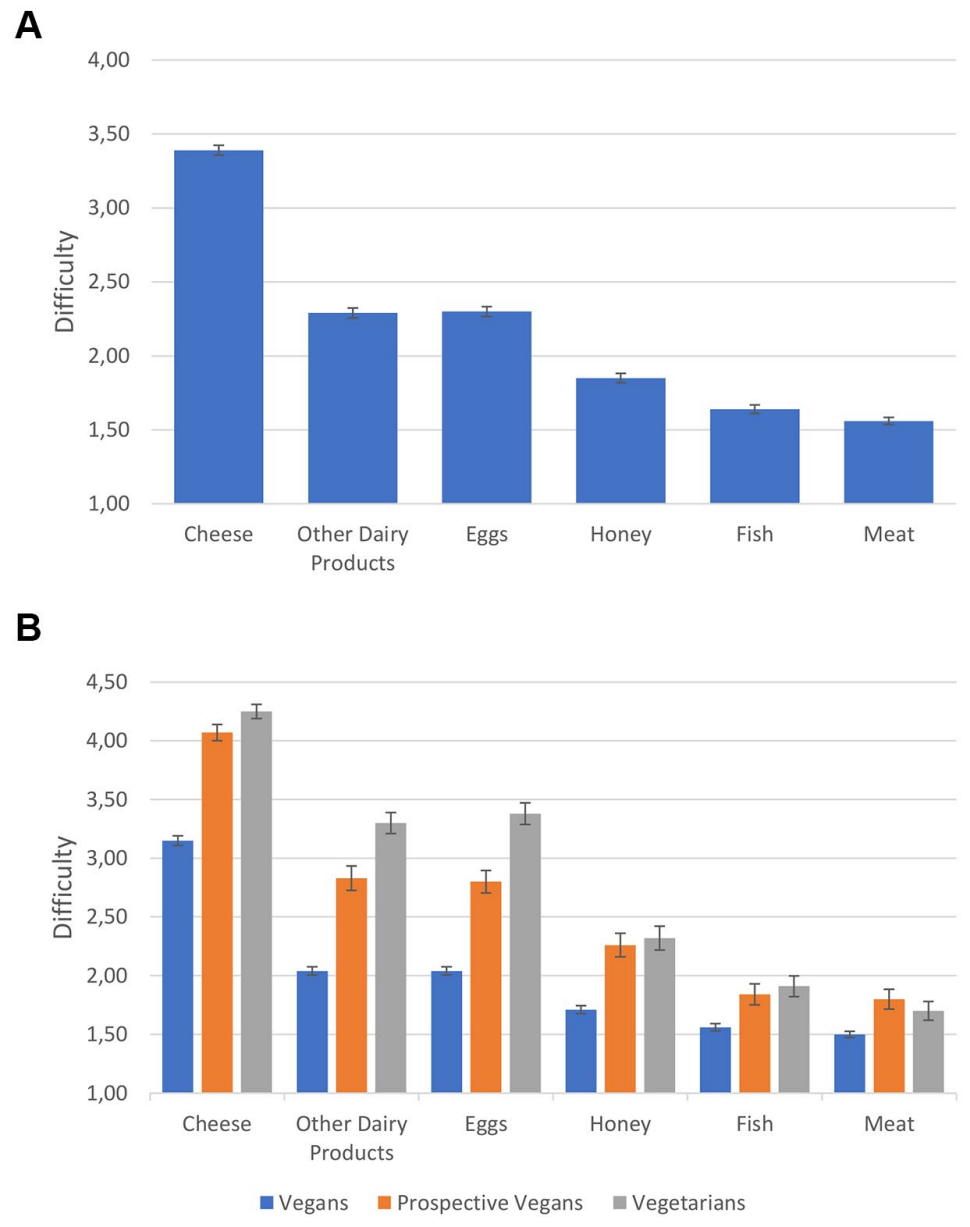
Difficulty of giving up certain types of food for all participants **(A)** and disaggregated by groups **(B)**. Error bars represent the standard error of the mean.

Figure 3 provides participants’ estimation of how difficult it actually was or would be to give up certain animal-derived food types.

A repeated measures ANOVA with food type as within factor and lifestyle as between factor shows that there were significant differences: First, the three lifestyles/groups differed significantly in their overall estimation of the difficulty of giving up animal-derived food: *F*(2, 1357) = 162.34, *p* < 0.001, *η_p_^2^* = 0.19. *Post hoc* analyses, using a Tukey-HSD test, revealed that all comparisons between groups were significant (vegans vs. vegetarians and vs. prospective vegans: *p*s < 0.001, vegetarians vs. prospective vegans: *p* = 0.008). Second, all comparisons between food types were significant: *F*s(1, 1356) > 148.52, *p*s < 0.001, η_p_^2^s > 0.10, with the exception of other dairy products vs. eggs, *F*(1, 1356) = 0.24, *p* = 0.622, η_p_^2^ = 0.01. Third, all interactions between lifestyle and food type were significant, all *F*s(1, 1356) > 3.59, *p*s < 0.028, η_p_^2^s > 0.01.

### 3.3 Knowledge and vegan literacy

Between all three groups, there were significant differences in participants’ self-assessment regarding knowledge of diets (vegans: *M* = 79.52, *SD* = 14.90; prospective vegans: *M* = 73.40, *SD* = 17.03; vegetarians: *M* = 72.64, *SD* = 17.52), knowledge of the animal industry (vegans: *M* = 89.17, *SD* = 14.60; prospective vegans: *M* = 84.47, *SD* = 15.44; vegetarians: *M* = 76.20, *SD* = 17.94), and general assessment of the animal industry (vegans: *M* = 6.07, *SD* = 18.05; prospective vegans: *M* = 7.23, *SD* = 16.81; vegetarians: *M* = 11.07, *SD* = 14.51). A MANOVA showed that there were significant differences between groups: self-assessment knowledge diets: *F*(2, 1417) = 24.06, *p* < 0.001, η_p_^2^ = 0.033, self-assessment knowledge animal industry: *F*(2, 1417) = 52.29, *p* < 0.001, η_p_^2^ = 0.076, assessment animal industry: *F*(2, 1417) = 6.25, *p* = 0.002, η_p_^2^ = 0.009. All Tukey-HSD *post-hoc* tests between groups were significant (*p*s < 0.001), with the exception of prospective vegans vs. vegetarians regarding knowledge of diet (*p* = 0.887) and assessment of the animal industry (*p* = 0.086), and vegans vs. prospective vegans for knowledge of the animal industry (*p* = 0.669). Furthermore, it is worth noting that among vegans, 67.3% of participants gave the lowest possible rating when assessing the animal industry, among prospective vegans 56.9% gave the lowest possible rating, and among vegetarians 30.5% gave the lowest possible rating.

**Figure 4:**
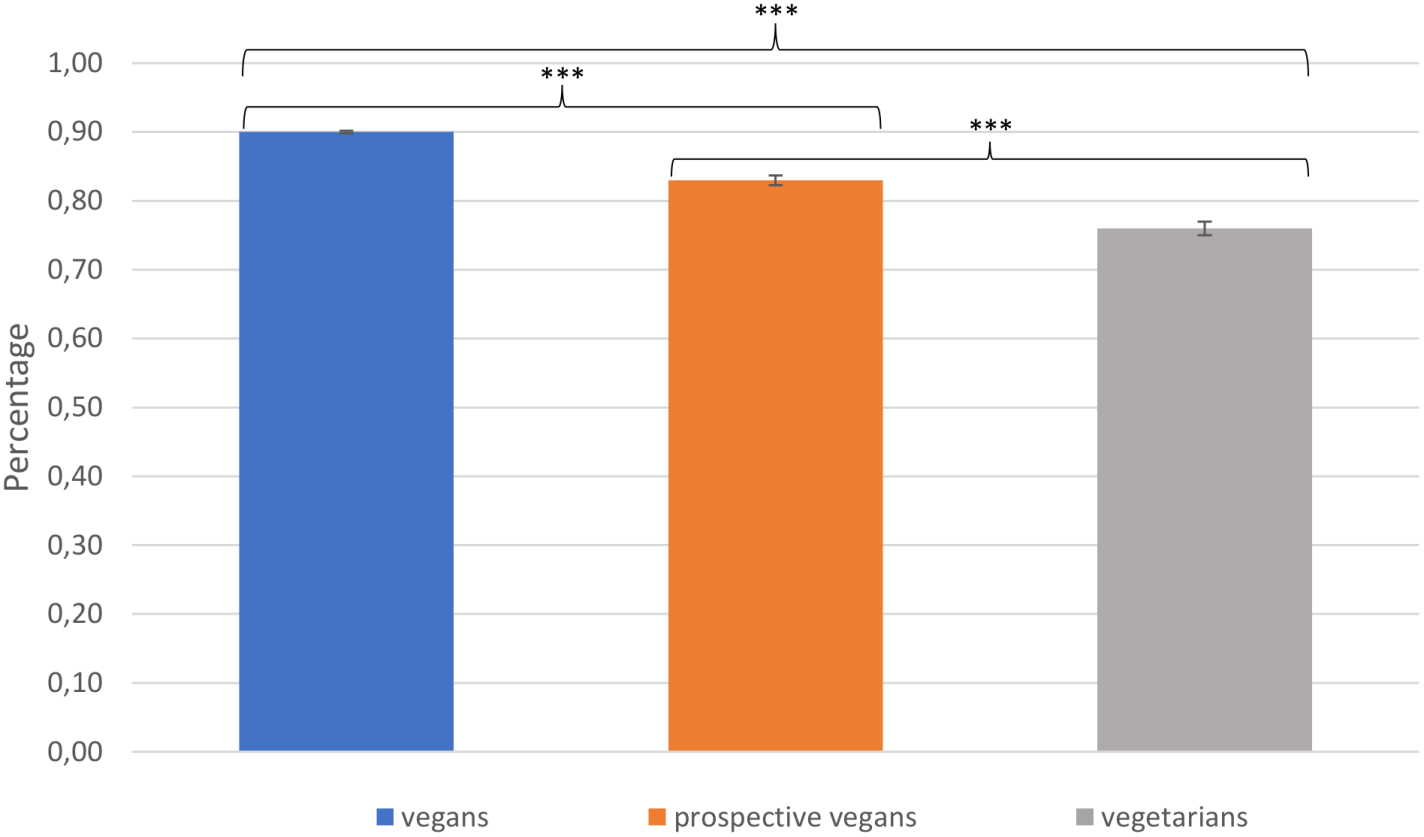
Communicative knowledge: percentage of correct answers.

For communicative knowledge (see Figure 4), i.e., for the percentage of correct answers, there were significant differences between the three groups: vegans scored highest (*M*_vegans_ = 0.90, *SD*_vegans_ = 0.08), followed by prospective vegans (*M*_prosp. veg._ = 0.83, *SD*_prosp. veg._ = 0.10) and vegetarians (*M*_vegetarians_ = 0.76, *SD*_vegetarians_ = 0.13): *F*(2, 1417) = 180.03, *p* < 0.001, *η_p_^2^* = 0.203. All *post-hoc* comparisons were significant: all *p*s < 0.001.

The important role of cheese can be illustrated by the observation that the item “Cheese is always vegetarian” (which is false) was correctly answered by 90.63% of the vegans, 87.82% of the prospective vegans, and 80.79% of the vegetarians. Similarly, the item “Not every cheese is vegetarian because some varieties contain rennet, which is derived from the stomach of calves” (which is correct) was correctly answered by 94.65% of the vegans, 92.89% of the prospective vegans, and 82.49% of the vegetarians. Finally, the item “A vegetarian diet means that no animals died for one’s diet” (which is false) was correctly answered by 91.40% of the vegans, 80.20% of the prospective vegans, and 62.71% of the vegetarians.

The participants’ own assessment of their knowledge about diets [*r*(1420) = 0.26, *p* < 0.001] and the animal industry [*r*(1420) = 0.32, *p* < 0.001] was significantly positively correlated with their communicative knowledge.

A Kruskal–Wallis test showed that time spent per month on information about diet, the animal industry, or animal welfare and/or animal rights differed significantly between vegans, prospective vegans, and vegetarians: *H*(2) = 120.95, *p* < 0.001, η_p_^2^ = 0.083. *Post hoc* Mann–Whitney tests revealed that all comparisons between pairs of groups were significant: all *p*s < 0.001. Vegans spent the most time per month on acquiring information, followed by prospective vegans. Vegetarians spent the least amount of time. Table 1 provides the respective percentages.

**Figure 5:**
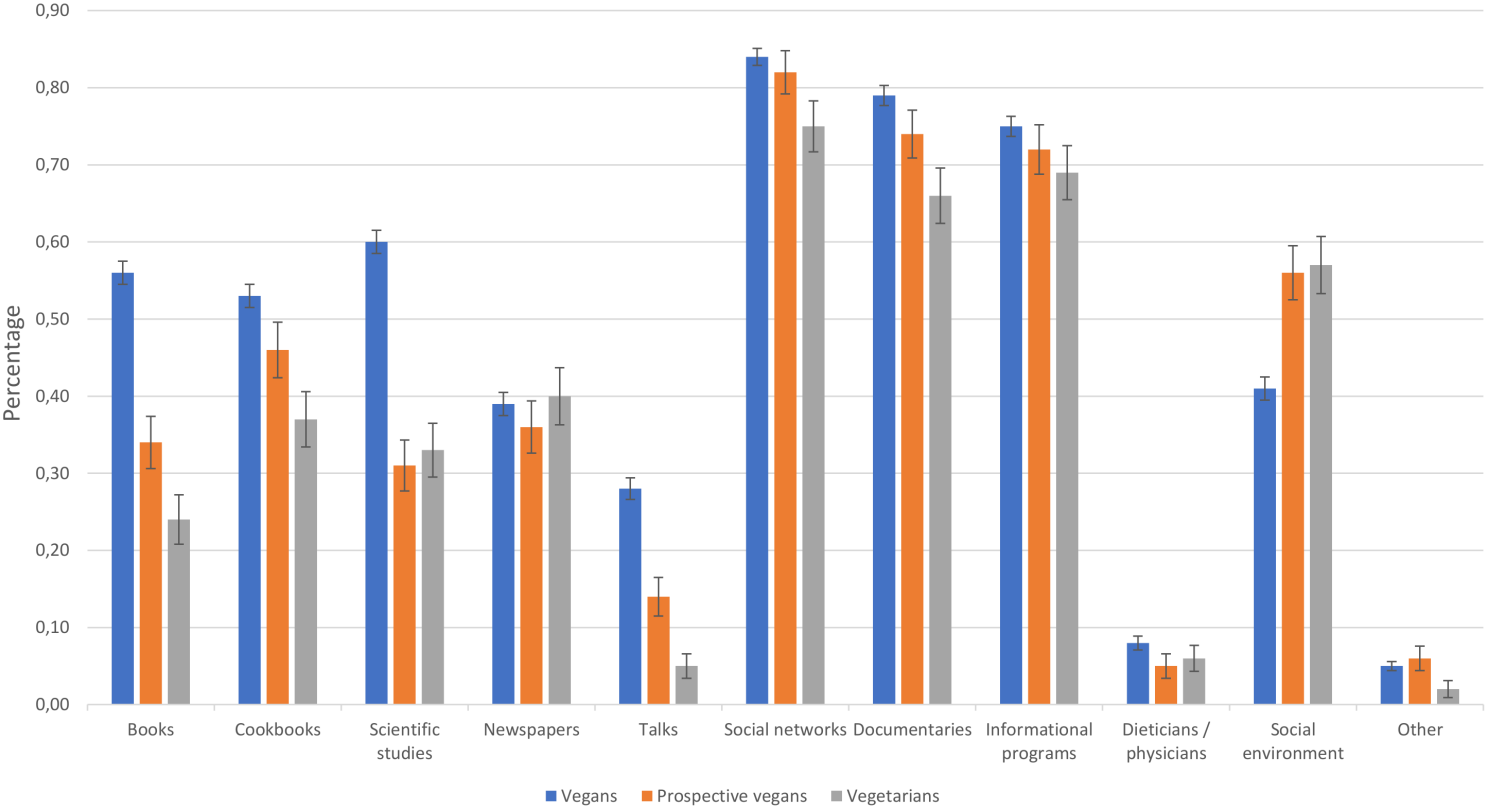
Sources of information: percentage of participants who stated they consulted appropriate source. Error bars represent the standard error of the mean.

A MANOVA showed that there are also significant differences in the sources of information between groups (Figure 5) as indicated by the percentage (dummy-coded) of how many participants stated that they consulted the appropriate source of information: books: *F*(2, 1417) = 43.82, *p* < 0.001, η_p_^2^ = 0.058, cookbooks: *F*(2, 1417) = 8.26, *p* < 0.001, η_p_^2^ = 0.012, scientific studies: *F*(2, 1417) = 45.57, *p* < 0.001, η_p_^2^ = 0.060, newspapers: *F*(2, 1417) = 0.41, *p* = 0.661, η_p_^2^ = 0.001, talks: *F*(2, 1417) = 29.08, *p* < 0.001, η_p_^2^ = 0.039, social networks: *F*(2, 1417) = 4.24, *p* = 0.015, η_p_^2^ = 0.006, documentaries: *F*(2, 1417) = 7.51, *p* = 0.001, η_p_^2^ = 0.010, informational programs: *F*(2, 1417) = 1.53, *p* = 0.216, η_p_^2^ = 0.002, dieticians/physicians: *F*(2, 1417) = 1.87, *p* = 0.155, η_p_^2^ = 0.003, social environment: *F*(2, 1417) = 13.21, *p* < 0.001, η_p_^2^ = 0.018, other: *F*(2, 1417) = 1.31, *p* = 0.270, η_p_^2^ = 0.002.

The fact that vegans rely significantly more on scientific studies than both prospective vegans and vegetarians cannot be attributed to different levels of education—which in turn could imply being more familiar with reading scientific studies—seeing as a Kruskal–Wallis test showed no significant differences in the level of education between the three groups: *H*(2) = 0.21, *p* = 0.898, *η_p_^2^* < 0.001.

### 3.4 Dietarian identity, attitude, affectivity, and instrumental knowledge

Furthermore, a MANOVA showed significant differences in dietarian identity, attitude, affectivity, and instrumental knowledge (Table 2).

**Table 2:** Dietarian identity, attitude, affectivity, and instrumental knowledge.

	Vegans		Prospective vegans		Vegetarians				
	*M*	*SD*	*M*	*SD*	*M*	*SD*	*F*(2, 1417)	*p*	η_p_^2^
Dietarian identity	3.34	0.50	3.34	0.48	3.08	0.55	20.36	<0.001	0.03
Attitude	4.19	0.23	4.00	0.26	3.96	0.28	97.37	<0.001	0.12
Affectivity	4.54	0.46	4.51	0.49	4.39	0.50	8.41	<0.001	0.01
Instrumental knowledge	4.38	0.39	3.87	0.60	4.20	0.55	301.55	<0.001	0.30

Tukey-HSD *post-hoc* tests showed that there were no differences between vegans and prospective vegans concerning dietarian identity (*p* = 0.994) or affectivity (*p* = 0.627), but both groups differed significantly from vegetarians (all *p*s < 0.032). As for attitude, vegans differed significantly from both prospective vegans and vegetarians (all *p*s < 0.001), while there was no difference between prospective vegans and vegetarians (*p* = 0.254). All comparisons between groups were significant for instrumental knowledge (all *p*s < 0.001).

A linear regression was calculated to explore whether dietarian identity, attitude, affectivity, communicative knowledge, and instrumental knowledge can predict how long vegans have been following their dietary pattern. The regression model was significant [*F*(5, 1040) = 9.75, *p* < 0.001, *R^2^* = 0.045], significant predictors being dietarian identity (*β* = −0.11, *p* < 0.001), attitude (*β* = 0.14, *p* < 0.001), and instrumental knowledge (*β* = 0.07, *p* = 0.017). The predictors affectivity (*β* = 0.02, *p* = 0.435) and communicative knowledge (*β* = 0.02, *p* = 0.405) did not reach significance.

Furthermore, comparing vegetarians who can envisage becoming vegan (potential vegans) to those vegetarians who cannot imagine the transition to a vegan diet, a MANOVA was conducted for dietarian identity, attitude, affectivity, instrumental knowledge and communicative knowledge, assessment of the participants’ own knowledge concerning diet and the animal industry, and assessment of the animal industry to discover which factor might facilitate the transition to a vegan diet. Significant differences were found for dietarian identity (*M*_potential vegans_ = 3.18, *SD*_potential vegans_ = 0.54, *M*_non-potential vegans_ = 2.88, *SD*_non-potential vegans_ = 0.55; *F*(1, 167) = 10.64, *p* = 0.001, η_p_^2^ = 0.060), assessment of the animal industry (*M*_potential vegans_ = 9.06, *SD*_potential vegans_ = 9.42, *M*_non-potential vegans_ = 17.81, *SD*_non-potential vegans_ = 22.35; *F*(1, 167) = 12.87, *p* < 0.001, η_p_^2^ = 0.072), and instrumental knowledge (*M*_potential vegans_ = 3.60, *SD*_potential vegans_ = 0.61, *M*_non-potential vegans_ = 3.37, *SD*_non-potential vegans_ = 0.67; *F*(1, 167) = 4.21, *p* = 0.042, η_p_^2^ = 0.025). Differences in attitude, affectivity, communicative knowledge, assessment of knowledge concerning diet and the animal industry were all not significant (all *p*s > 0.055).

## 4 Discussion

The aim of the present study was to investigate whether and how vegans, prospective vegans, and vegetarians differ on a psychological level to explain why a similar motivation, namely, the desire not to harm animals and to protect the environment, leads to different behaviors. The presented data suggest that, on the whole, vegans, vegetarians, and prospective vegans pursue similar goals and motivations regarding their lifestyle choices. However, seeing as there is a significant difference in knowledge between vegans and vegetarians, with prospective vegans located between these two groups, the existing differences in behavior may be attributed to differences in knowledge between these groups, therefore leading them to draw different conclusions regarding their eating behavior.

Regarding knowledge, the presented data clearly portray that groups differ: The most important finding shows that vegans have significantly more knowledge compared to vegetarians and prospective vegans on the effects of vegan and vegetarian diets on animal-related, environmental, and health issues, i.e., communicative knowledge, according to McDonald (2000). This provides important insights with regard to motivation. If all three groups assess animal rights and animal welfare and the environment as important factors, the gap between why some participants chose a vegan diet and others a vegetarian diet—based on similar objectives—may be explained by the role of knowledge. Appropriately, vegetarians valuate the animal industry significantly less negatively than vegans and prospective vegans, which supports this argument. Moreover, the results show that vegetarians generally possess less correct information about the animal industry—as, for example, indicated by the fact that a third of the vegetarians were not aware that their dietary choices still lead to the death of animals.

Fittingly, vegans spend more time acquiring information about diet and animal-related issues compared to the other groups and also more frequently rely on reputable sources, such as scientific studies. This difference may be explained by the fact that learning about veganism and related topics is a key component in becoming vegan (MacNair, 2001). This would also explain why prospective vegans are once again located between the other two groups. Interestingly, the only source of information where vegans do not score highest is social environment, implying that the only source of information accessed more frequently by non-vegans is a source that is less reliable and less capable of providing objective information.

The finding showing that taste is more important to vegetarians than to vegans and prospective vegans seems rather counterintuitive. However, it is unlikely that taste is less important to vegans, and therefore, this finding may rather be a reflection of vegetarians’ seeking to express the importance of their current lifestyle choices. This implies that vegetarians’ placing emphasis on taste could be an affirmation of their current eating habits, possibly linked to a certain unwillingness to give up animal products. Similarly, the finding stating that it may be difficult for vegetarians not to consume dairy products—especially cheese—and eggs because they would miss the taste and “substitute” products often fail to meet consumers’ taste expectations (Docherty and Jasper, 2023) implies that a vegan lifestyle may be associated with the expectation of a worse taste experience. This finding is confirmed by the results of this study, and especially the important role of cheese is highlighted. Consequently, vegetarians may be discouraged from adopting or maintaining a vegan lifestyle. A similar reasoning could apply to habit, which does not play a significant role for vegans compared to vegetarians and also to prospective vegans. The present results suggest that both non-vegan groups rate the role of habit highly, possibly because they are aware of the fact that they may find it difficult to change their dietary habits. This was already proposed by Pohjolainen et al. (2015), who reported that people generally have difficulties changing their eating behaviors. Merging these last two points, the data of this study suggest that vegetarians and possibly also prospective vegans have acquired a sense of comfort regarding their current lifestyle.

Matching this rationale, the data showed that—when exploring why vegetarians choose to stay vegetarian—taste once again plays an important role, most prominently so with regard to cheese. Approximately half of the vegetarians cannot imagine abstaining from animal products due to their taste. This is consistent with findings that one of the main reasons for a vegetarian diet is taste (Beardsworth and Keil, 1991). However, cheese seems to be a rather unique case where knowledge plays a crucial role. The present analysis clearly shows a gap in knowledge regarding the question of whether or not cheese is vegetarian. Therefore, vegans appear to be more literate when it comes to factual knowledge concerning animal welfare, environmental issues, and veganism, thus basing their dietary choices on this knowledge. This matches the research stating that acquiring knowledge is an indispensable part of transitioning to a vegan lifestyle (e.g., MacNair, 2001). The term “food literacy” is used to describe “the everyday practices associated with navigating the food system” (Vidgen and Gallegos, 2014, p. 51; see also Groufh-Jacobsen et al., 2023 for a recent discussion). This approach translates to the present study by employing the term “vegan literacy,” which describes a cluster of knowledge regarding veganism. In our understanding, vegan literacy entails knowledge about diet, lifestyle, animal rights and welfare, and environmental issues. In summary, vegan literacy comprises both the knowledge needed to follow a vegan lifestyle in everyday life and the effect that a vegan lifestyle has on animals, health, and the environment.

Although it was or would be (if cheese was still part of the participants’ diet) significantly harder to give up cheese than other animal-derived foods for all groups, prospective vegans and vegetarians indicated greater difficulties than vegans, in turn supporting the crucial role of cheese—especially for vegetarians and potential vegans. Some of the participants even described their inability to resist cheese as addiction-like (“Cheese is addictive […].,” “Craving, I’d say. Just like a dry alcoholic.”; see also similar statements as reported in Docherty and Jasper, 2023). The results also corroborate the findings discussing the potentially addictive nature of cheese (Schulte et al., 2015) and evidence for food addiction more globally (Gordon et al., 2018). Naturally, this topic requires further detailed investigation. However, linking the issue of cheese to the question of knowledge, the present study found that vegetarians actually know less about cheese and the fact that its production is often not vegetarian than vegans and prospective vegans, further highlighting the differences in knowledge between these groups. It has been suggested that differences with regard to moral concepts about animal products (Weber and Kollmayer, 2022) or psychological mechanisms such as cognitive dissonance (Docherty and Jasper, 2023; Ioannidou et al., 2023) might explain why consumption of non-meat animal products, such as cheese—the so-called “cheese paradox”—is perceived as unproblematic by many. In light of these findings, these explanations should be complemented by the crucial role of knowledge.

Health is also addressed in the present study: The finding that health is less important to vegetarians than to vegans and prospective vegans may also be interpreted in the context of knowledge: As knowledge and information seeking are central factors in becoming vegan, an in-depth engagement with information on veganism and how veganism affects health may increase peoples’ general health awareness. This train of thought would also match the position of prospective vegans between vegans and vegetarians.

Sustainability and ecological reasons are most important for prospective vegans, which could highlight why this group intends to become vegan in the future, having learned that a vegan lifestyle has the smallest ecological footprint (Notarnicola et al., 2017; Chai et al., 2019; Rabès et al., 2020; Allenden et al., 2022; Filippin et al., 2023; McDougall, 2023; Scarborough et al., 2023). Once they have acquired more information about a vegan lifestyle, they will, as implied by the work of McDonald (2000), become vegan.

Animal rights and animal welfare are most important to vegans (Rothgerber, 2015a) compared to the other groups, which could explain why vegetarians did not express the aim to become vegan. Exploring the question of why prospective vegans have not taken the final step of becoming vegan, the present data suggest that this also could be linked to knowledge, implying that prospective vegans may have carried out less extensive research on the conditions in the animal industry, resulting in less knowledge about these conditions.

The presented data also show that vegans have more knowledge and acquire more information from scientific studies compared to the other groups. Appropriately, prospective vegans—assuming that they had already engaged with the subject matter while considering transitioning to a vegan lifestyle—named animal rights and welfare as one of the crucial reasons for intending to become vegan in the future. However, the data illustrate that all three groups are motivated in their lifestyle choices by wanting to protect animals. On the other hand, the data reveal a significant effect differentiating vegans and non-vegans regarding animal rights and welfare. Furthermore, the most important motivation for prospective vegans to become vegan is linked to environmental issues. This result matches the findings indicating that environmental and climate issues and their relation to dietary choices are topics which especially young people are highly aware of (Jürkenbeck et al., 2021; Venghaus et al., 2022). This suggests that the final nudge to become vegan is rather due to environmental issues than to animal-related topics, even though these also play an important role in the process of becoming vegan. Furthermore, not to be overlooked is the fact that a substantial number of vegetarians and prospective vegans assume that a vegetarian diet is already the best choice as far as animal rights and welfare are concerned—a finding which, in addition to highlighting once again the prominent role of knowledge, may also, *inter alia*, help to explain vegetarians’ reluctance to transition to a vegan diet.

Regarding the social environment, the data revealed striking differences between groups. This matches the prevailing literature, indicating that a lack of social support for a vegan lifestyle is a major hindrance to the adoption and retention of said lifestyle (e.g., Haverstock and Forgays, 2012; Markowski and Roxburgh, 2019; Sirieix et al., 2023). However, previous studies indicate that the social environment’s influence seems to be more important to vegetarians than to vegans (Kessler et al., 2016; Rosenfeld and Tomiyama, 2019; see also Sirieix et al., 2023). This would back up the results of the present study, which show that vegans rated the factor “social environment” as less important than the ratings allocated by vegetarians and prospective vegans. This indicates that social environment mainly becomes a crucial factor if it is perceived as a hindering factor. Furthermore, this effect could be a result of peoples’ fear of stigmatization by their social surroundings should they decide to adopt a vegan lifestyle, as already suggested by previous studies (MacInnis and Hodson, 2017; Judge and Wilson, 2019; Markowski and Roxburgh, 2019).

Feasibility in everyday life is least important to vegans, followed by prospective vegans and vegetarians. This may highlight why vegetarians decide not to adopt a vegan diet and also may be a reflection of prospective vegans’ concerns regarding said transition to veganism, aligning with previous studies (Cole and Morgan, 2011; Giacoman et al., 2023). Based on the present data, the aspect of feasibility seems to be one of great importance, especially for vegetarians and prospective vegans who envisage becoming vegan to be relatively difficult. As vegans have previously implemented their vegan lifestyle, they have already experienced that adherence to veganism is possible and feasible in everyday life.

Addressing dietarian identity, the present data indicate that dietarian identity is more important to vegans and prospective vegans than to vegetarians, as has already been proposed (Rosenfeld, 2019; Kirsten et al., 2020). The dietarian identity of prospective vegans has a significance similar to that of vegans. This may be attributable to the fact that prospective vegans have already researched their diet, and therefore, the concept of their dietarian identity is more salient compared to that of vegetarians.

Regarding affectivity, vegans and prospective vegans display a higher level of affectivity toward animals and toward the environment than is displayed by vegetarians. This corresponds to previous findings (Rothgerber, 2015a). As already established above with regard to other concepts such as dietarian identity, prospective vegans were located between vegans and vegetarians. Moreover, referring to attitude the data portrayed that the two non-vegan groups are more similar to one another in this regard than they are concerning previous measures. This indicates that both vegetarians and prospective vegans have not (yet) internalized some of the fundamental components of veganism. Regarding instrumental knowledge, which in this context may be interpreted as following a vegan lifestyle in everyday life, vegans—unsurprisingly—showed the most competence and feasibility. Fittingly, prospective vegans are located between vegans and vegetarians.

Furthermore, dietarian identity, attitude, and instrumental knowledge were significant predictors for the duration of a vegan diet, whereas affectivity and communicative knowledge were not significant. This may be attributed to the high level of knowledge within the group of vegans, indicating a ceiling effect. However, dietarian identity was—unexpectedly—a negative predictor, suggesting that a lower dietarian identity predicted a longer duration of a vegan lifestyle. This can be interpreted as an indication that the longer an individual has already been vegan, the less this is necessarily perceived as a central part of one’s identity, meaning that it may be natural and/or unthinkable to live any other way. Although no explanations are suggested by the existing literature, the finding that affectivity does not predict a longer duration of a vegan lifestyle may be attributed to the fact that research has mainly focused on affectivity in the context of *becoming* and not of *staying* vegan, therefore attributing a catalytic effect to affectivity in the process of transitioning to a vegan diet (e.g., McDonald, 2000; Salehi et al., 2020).

According to McDonald’s (2000) framework, individuals change due to a catalytic experience (e.g., watching a video addressing cruelty in the animal industry; also proposed by Salehi et al., 2020). Hereafter, individuals can either become more oriented toward a vegan or vegetarian lifestyle or repress this information. In addition, many people seem to be aware of some details of the cruelty involved in animal farming but repress thinking about these issues (Bryant et al., 2022), thus maintaining the status quo, indicating that vegetarianism may represent an intermediate stage in which individuals are already aware of the fact that the animal industry harms animals (therefore resulting in the renunciation of meat), while at the same time potentially not wishing to know all the details about, for example, the production of cheese, which could then result in a broader change in lifestyle.

The next step in (potentially) transitioning to a vegan lifestyle is learning about veganism and pertinent topics, such as animal abuse or cooking vegan meals (McDonald, 2000). Furthermore, becoming vegan or vegetarian entails processing said information on both the emotional and the cognitive level (Salehi et al., 2020), highlighting the important role of knowledge in the process of becoming vegan or vegetarian.

### 4.1 Limitations and implications for further research

Concerning the results of the present study, it may be beneficial to further investigate the role of gender. The majority of the sample in the present study was female, which indicates, on the one hand, that the sample was representative for vegans and vegetarians in general, thus ensuring ecological validity, because it is a well-established fact (e.g., Ruby, 2012; Modlinska et al., 2020) that the majority of vegetarians and vegans are female, at least in Western cultures. On the other hand, it is also well-known that both veganism and vegetarianism are strongly affected by gender-related issues, such as the perspective or opinion that being vegan or vegetarian is considered “unmanly” (Thomas, 2016; Modlinska et al., 2020; Rosenfeld, 2020). Therefore, further research might elucidate whether or how gender affects the findings of this study.

As already outlined, it could be particularly interesting to investigate the potentially addictive nature of cheese, seeing as cheese may be a crucial factor when it comes to becoming vegan or remaining vegetarian. Furthermore, it remains unclear why vegetarians have less knowledge than vegans, even though both groups pursue similar goals, therefore portraying similar motives in their dietary choices. The fact that vegans spend more time on acquiring knowledge than vegetarians, additionally knowledge from more reliable sources, suggests that vegans are also more motivated to invest more time and effort in acquiring said knowledge. However, the question of why they are more motivated to do so remains to be answered.

Since perceptions and cognitions pertaining to vegan and vegetarian lifestyles seem to differ between (Western) countries, e.g., between the USA and the Netherlands (Zaal et al., 2023), it is also necessary to investigate to what extent the present findings are applicable beyond the German cultural sphere, seeing as countries differ with regard to the range of available vegan products, restaurant offers, etc. This influences the formation of not only opinions and perceptions of veganism but also, potentially, feasibility in everyday life.

On a more general level, there is still a comparatively limited bandwidth of research on the differences and similarities between vegans, prospective vegans, and vegetarians. There are already studies addressing this issue for ecology (Filippin et al., 2023) and sports and health (Wirnitzer, 2020), but especially in psychology, more research is necessary to elucidate the differences between vegans, prospective vegans, and vegetarians, which are, after all, rather comprehensive sets of behavior. Finally, it is important to note that all results and conclusions of the present study are based on an online survey and not on an experiment, which means that, evidently, causal relationships cannot be established and the results should be regarded as exploratory. The aim of this study was to comprehensively identify psychological differences between vegans, prospective vegans, and vegetarians. To this end, a number of variables were investigated but, given the tentative nature of these findings, further research from different methodological angles and with a focus on the specific workings of each factor found in the present study is required to corroborate the results of the present study.

## 5 Conclusion

In conclusion, a multitude of factors may influence whether an individual chooses to transition to a vegan diet, including, among other reasons, concern about animals and the environment, attitude and social environment, or affectivity. However, the most important factor in becoming vegan appears to be knowledge. The significance of the present study lies in the fact that the results as presented go beyond the role of knowledge merely with regard to changing dietary habits, thus extending the so far existing scope of research and highlighting how knowledge is an essential factor in differentiating vegetarians from vegans on a psychological level.

Overall, the presented data show a consistent pattern, indicating that vegans know significantly more about animal issues, such as the conditions in the animal industry. In addition, they have more overall factual knowledge concerning diet, therefore emphasizing the importance of vegan literacy in the question of whether to become vegan or remain vegetarian. Considering that many people express the desire to choose a lifestyle which harms animals and the environment as little as possible, increased availability of reliable information concerning appropriate topics (animal industry, diet, environmental and sustainability issues, etc.)—i.e., higher vegan literacy—is likely to support such a transition. In addition, this lack of knowledge on the part of vegetarians may also lead to vegetarians’ sense of comfort and habit which seems to accompany their dietary choices. Another component making up the difference between vegans and vegetarians (with prospective vegans consistently located between the other two groups, therefore illustrating their transitory position between different lifestyles) comprises factors such as taste—and most prominently: cheese—and feasibility in everyday life. This corresponds to the role of knowledge, habit, and a certain reluctance to change displayed by vegetarians, seeing as they do share many of the ethical and motivational aspects cited by vegans (i.e., animal welfare, protecting the environment, etc.) without translating—or fully translating—these into action.

Finally, the present data indicate that both vegans and prospective vegans have already delved deeper than vegetarians into the subject matter of how lifestyle and dietary choices are linked to the environment, health, and animal-related issues—therefore leading to the conclusion on their part that veganism represents the lifestyle choice which matches the knowledge they have acquired and is in harmony with their ideals.

## Data availability statement

The raw data supporting the conclusions of this article will be made available by the authors, without undue reservation.

## Ethics statement

Ethical approval was not required for the studies involving humans because according to German law, no ethical approval is required as there was no potential harm for participants. The studies were conducted in accordance with the local legislation and institutional requirements. The participants provided their written informed consent to participate in this study.

## Author contributions

RM: Conceptualization, Design, Data collection, Data analysis, Writing. LR: Conceptualization, Design, Data collection, Data analysis, Writing. JH: Conceptualization, Design, Data collection, Data analysis, Writing. KF: Conceptualization, Design, Data collection, Data analysis, Writing.

## Footnotes

^1^Initially, it was planned to include former vegans in the analysis. However, considering that this group comprised only 12 participants, this group was excluded from all data analyses.
